# CAZac: an activity descriptor for carbohydrate-active enzymes

**DOI:** 10.1093/nar/gkae1045

**Published:** 2024-11-11

**Authors:** Vincent Lombard, Bernard Henrissat, Marie-Line Garron

**Affiliations:** Aix Marseille Univ, CNRS, UMR7257, INRAE, USC1408, AFMB, 163 Avenue de Luminy, 13288 Marseille, France; Aix Marseille Univ, CNRS, UMR7257, INRAE, USC1408, AFMB, 163 Avenue de Luminy, 13288 Marseille, France; Department of Biotechnology and Biomedicine (DTU Bioengineering), Technical University of Denmark, Søltofts Plads, 2800 Kgs. Lyngby, Denmark; Aix Marseille Univ, CNRS, UMR7257, INRAE, USC1408, AFMB, 163 Avenue de Luminy, 13288 Marseille, France

## Abstract

The Carbohydrate-Active enZYme database (CAZy; www.cazy.org) has been providing the reference classification of carbohydrate-active enzymes (CAZymes) for >30 years. Based on literature survey, the sequence-based families of CAZymes are enriched with functional data by using the International Union of Biochemistry and Molecular Biology Enzyme Commission (EC) number system. However, this system was not developed to search or compare functional information. To better harness functional information, we have developed CAZac (CAZyme activity descriptor), a multicriterion system that describes CAZymes’ mechanisms, glycosidic bond orientations, subsites and inter-residue connectivities. This new system, implemented for glycoside hydrolases, glycoside phosphorylases, transglycosidases, polysaccharide lyases and lytic polysaccharide monooxygenases allows complex searches in the CAZy database to uncover the evolution of substrate specificity and mechanisms of CAZymes across families.

## Graphical Abstract

**Graphical Abstract:**
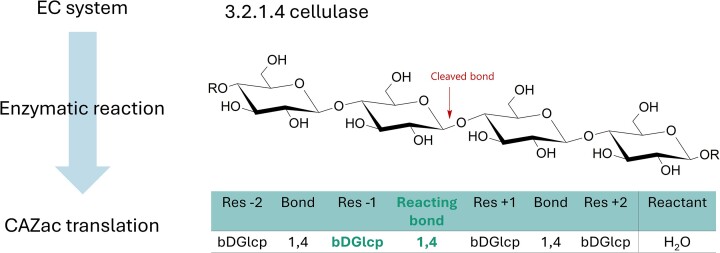

## Introduction

Present in all living organisms, carbohydrates are building blocks of life. Involved in structure, energy storage and a plethora of intra- and intercellular signaling cascades including host–pathogen interactions, the diversity of glycoconjugates, oligo- and polysaccharides is enormous. Carbohydrate-active enzymes (CAZymes) designate several classes of enzymes that assemble, breakdown or modify oligo- and polysaccharides. Because of the multiple roles of carbohydrates in nature, these enzymes find numerous applications in health, nutrition and biotechnology. With the growing availability of sequence data, a sequence-based family classification was initiated in 1991 for glycoside hydrolases (GHs) and subsequently extended to other CAZyme classes (1). Since 1998, the classification of CAZymes is presented in the continuously updated Carbohydrate-Active enZYme database (CAZy; www.cazy.org) (2). The CAZy database is currently organized around five functional groups, namely GHs, polysaccharide lyases (PLs), auxiliary activities (AAs), glycosyltransferases (GTs) and carbohydrate esterases. Of these different classes, GHs, PLs and a particular subclass of AAs described as lytic polysaccharide monooxygenases (LPMOs) catalyze glycosidic bond cleavage.

To standardize enzyme nomenclature and to avoid the proliferation of trivial names, the International Union of Biochemistry and Molecular Biology proceeded to classify and designate all the enzymatic activities using a system known as the Enzyme Commission (EC) numbers (3) that are organized in a hierarchical structure comprising four levels. The first level describes the broad reactions: 1. oxidoreductases, 2. transferases, 3. hydrolases, 4. lyases, 5. isomerases, 6. ligases and 7. translocases. The second, third and fourth levels provide more information about the chemical bonds, groups or substrates that are involved in the reaction. In this system, GHs receive EC number 3.2.1.*x*, with ‘3.’ for hydrolases, ‘2.’ for glycosylases and ‘1.’ for glycosidases, i.e. enzymes that hydrolyze O- and S-glycosidic bonds. Besides hydrolysis, several GHs are also able to catalyze the reverse reaction, i.e. synthesize glycosidic bond by phosphorylation or transglycosylation. The EC annotation of these latter functions places them in the same category as GTs, with EC numbers specifying sugar transferred: 2.4.1.*x* for hexosyltransferases, 2.4.2.*x* for the pentosyltransferases and 2.4.3.*x* for the sialyltransferases. PLs receive EC numbers 4.2.2.*x*, since these enzymes catalyze glycosidic bond cleavage by β-elimination. Finally, LPMOs are assigned EC numbers 1.14.99.*x*, which correspond to oxidases using O_2_ even though they were subsequently shown to be essentially peroxygenases. In the EC system, the glycosidic bond-cleaving activities described above only designate with the fourth digit of the EC number the actual glycoconjugate, oligo- or polysaccharide undergoing catalysis. With progress in product analysis and clarification of mechanisms, several EC numbers have become obsolete. Currently (release: 5/9/2024), there are 226 EC numbers for GHs (including 21 obsolete numbers), 29 EC numbers for PLs (including 1 obsolete) and 4 EC numbers for LPMOs. Each EC number is associated to a name, often an historical name, which unfortunately has not evolved since its creation and can sometimes be ambiguous, such as β-amylase (3.2.1.2) created in the 40s (4). For most CAZymes, these names provide only limited information about the function (e.g. the catalytic mechanism is usually not specified) and the reading of the exact EC definition is necessary to understand which exact bond is cleaved in a complex glycan substrate. Compared with the stereochemical diversity of glycans, estimated to range between several thousands and billions (5), the number of EC numbers available for GHs, PLs and LPMOs is largely insufficient to cover that range and does not even cover all the activities reported in the literature. To partially fill this gap and to describe activities not yet covered by the EC nomenclature, the CAZy database includes ∼100 incomplete EC numbers, denoted ‘3.2.1.-’ for GHs, ‘4.2.2.-’ for PLs and ‘1.14.99.-’ for LPMOs. Currently, the CAZy database uses a total of ∼400 activities to describe these three categories of enzymes. Although extremely useful, the EC classification is essentially a listing of functions with no implied relationship between the different numbers that appear in the fourth digit, which is only based on the next available number in the list. The EC system allows searching CAZy families by EC number, but cannot be used to explore related functions that may result from divergent or convergent evolution. For example, the cleavage of the α-1,4 bond between two glucose residues is found in >10 different EC numbers. Because most sequence-based families of GHs group together enzymes that act on different substrates or generate different products, the comparison of different function within the same family is not possible without an advanced knowledge of the EC classification and of the variety of underlying molecular mechanisms. In consequence, the wealth of functional information on CAZymes is currently underexploited. To circumvent this limitation, we have developed a new CAZyme activity descriptor, called CAZa , to describe the main functions of CAZymes involved in glycosidic bond cleavage (GHs, PLs and LPMOs) and assembly (GTs, phosphorylases and transglycosylases). Here, we present the CAZac system implemented for the GHs, PLs and LPMOs. This system represents a powerful tool to display, search and compare functional information across CAZyme families.

## The various parameters in CAZac

To elaborate a system that applies to all glycan-modifying enzymes, we developed two types of parameters: (i) those that are valid across an entire sequence-based family such as the catalytic mechanism and the orientation of the glycosidic bond undergoing catalysis, and (ii) those that can vary from one enzyme to another and that describe the substrate, the product and the exact connections of the glycosidic bonds.

### Descriptors at the family level

#### Mechanisms

**Figure 1.**
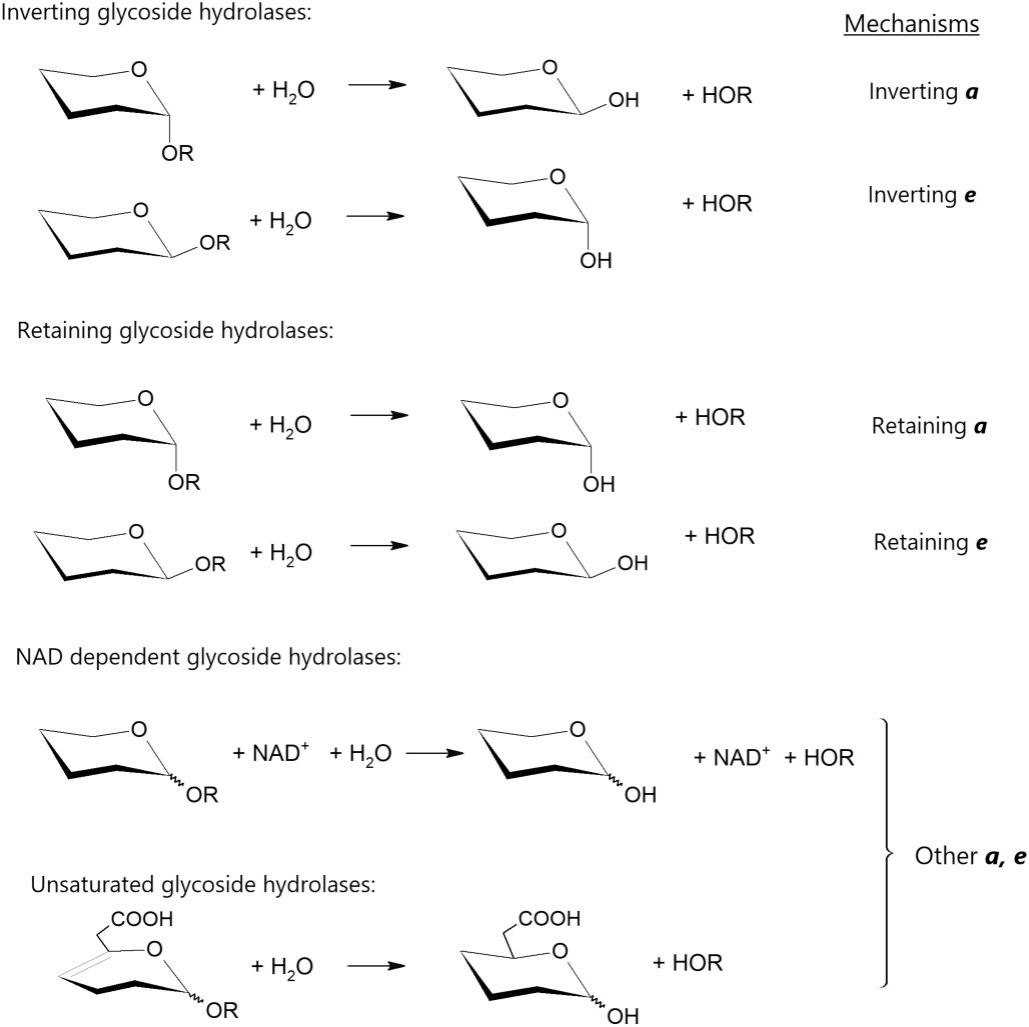
GH mechanisms. The orientation of the glycosidic bond is indicated by the letters ***a*** or ***e***, respectively, for axial or equatorial (13).

The first essential descriptor is the catalytic mechanism, which is missing from most GH EC numbers. Indeed, GHs adopt different mechanisms that are almost perfectly conserved within the sequence-based families as well as within the clans that group families with similar folds and a conserved catalytic machinery (6). The two main mechanisms of GHs have long been described by Koshland (7) and are based on the anomeric configuration of the carbon engaged in the glycosidic bond undergoing catalysis. Depending on the number of nucleophilic substitutions occurring at this carbon, the configuration is either conserved (retaining mechanism) or inverted (inverting mechanism) in the reaction product (Figure 1). Only one family of classical Koshland GHs, namely GH97, harbors both retaining and inverting GHs (8). Other mechanisms have been occasionally found such as in families GH4, GH109, GH177, GH179 and GH188, which contain nicotinamide adenine dinucleotide (NAD)-dependent enzymes (9,10), or families GH88 and GH105, which catalyze hydrolysis following hydration of the double bond between carbons C-4 and C-5 in their substrates (Figure 1) (11,12). Based on these various mechanisms, we defined five mechanistic categories for the GHs families: (i) retaining, (ii) inverting, (iii) retaining and inverting, (iv) other and (v) unknown for families for which the mechanism has not yet been established.

For GTs, the carbohydrate transferred from the activated donor to the acceptor also leads to the retention or inversion of the anomeric configuration of the glycosidic bond. Thus, the mechanism description of GT families follows the same denomination ‘retaining’ or ‘inverting’ as described above for GHs. The mechanisms of PLs and LPMOs are simply described by β-elimination and peroxygenase, respectively.

#### Stereochemistry of the anomeric carbon

The second essential parameter that is conserved at the family (and clan) level is the orientation of the glycosidic bond. CAZac specifies this orientation using three options: (i) axial, (ii) equatorial or (iii) axial and equatorial (Figure 1). The notation axial/equatorial was preferred over traditional α/β, as it represents the orientation of the bond regardless of the d/l stereochemistry of the sugar (13). For example, β-d-glucosides have an equatorial glycosidic bond just as α-l-arabinosides. The mixed category ‘axial and equatorial’ was created to account for families that are able to cleave both anomeric configurations through their specific NAD-dependent mechanism (9).

In summary, the two parameters above are used to describe, for each family, the mechanism of glycosidic bond-cleaving enzymes: retaining ***e***, retaining ***a***, inverting ***e***, inverting ***a***, other ***a*,*e*** including NAD-dependent families (GH4, GH109, GH177, GH179 and GH188) and the unsaturated GHs (GH88 and GH105), retaining and inverting ***a*** (GH97), β-elimination and peroxygenase (Figure 1).

### Descriptors for individual enzymes

It has long been observed that sequence-based CAZyme families group together enzymes of different substrate specificity and sometimes even different functions such as transglycosidases, phosphorylases, lyases or mutases. Thus, we have elaborated a system that would allow an accurate description of the main classes of CAZymes, their substrates and reaction products with a limited number of fields.

#### Subsites and activities

**Figure 2.**
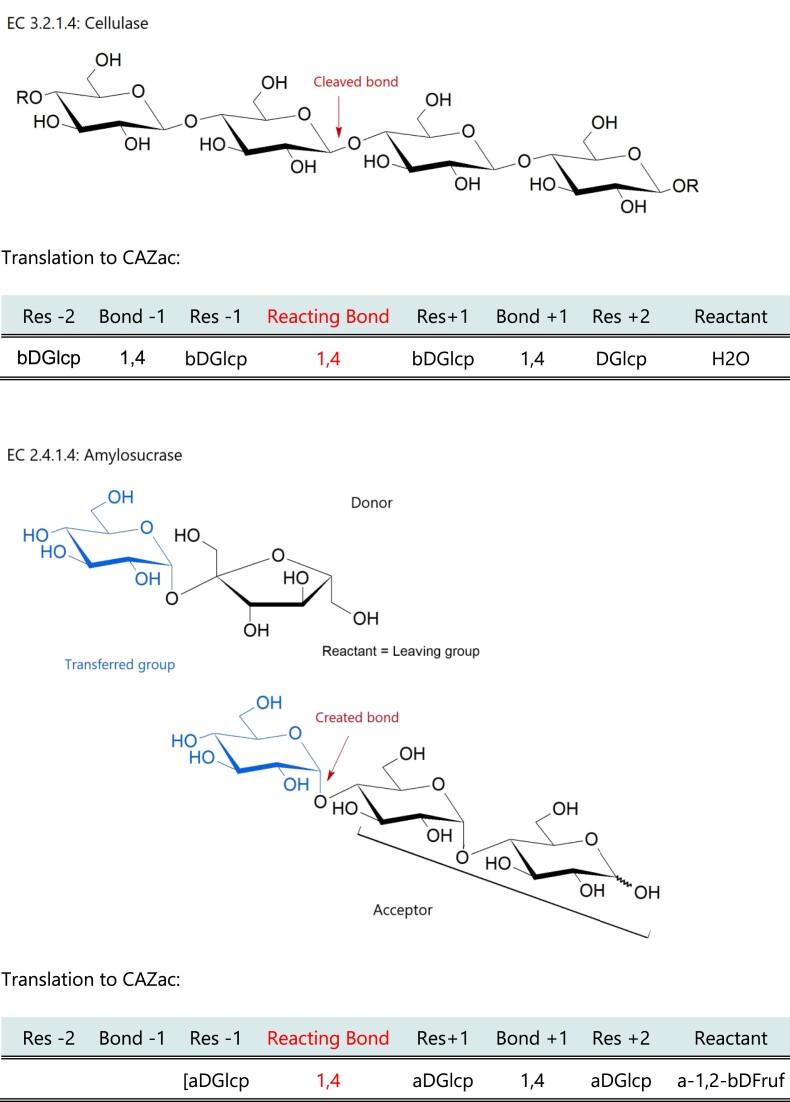
Translation of the enzymatic reaction in CAZac nomenclature. The transferred glucose residue is shown on the left.

The CAZac system follows the classical convention whereby glycans are oriented left-to-right from their nonreducing end (NRE) to their reducing end (RE), each monosaccharide residue filling a subsite in the enzyme active site. By convention (14), the subsites are numbered negatively and positively across the point of cleavage, with cleavage occurring between subsites −1 and +1, generating a novel RE for the monosaccharide in the −1 subsite. The description of GH activities is thus centered around the carbohydrate residue in the −1 subsite. By contrast, for PLs, the residue that undergoes catalysis is that at subsite +1 leading to a 4,5-unsaturation at the newly created NRE (15). In order to correctly describe CAZyme activities, we have opted to use eight fields including four subsites (−2, −1, +1 and +2), the three interconnecting glycosidic bonds and the reactant (Figure 2). The four subsites and the interconnecting bonds describe the chemical structure of the substrate for cleavage reactions, or the reaction product for transferases and mutases. The bond between subsites −1 and +1 is the cleaved or the created bond, and we term this bond the ‘reacting bond’. Finally, the reactant is the additional compound involved in the enzymatic reaction. The addition of this field was motivated to describe the transglycosylating activities of GHs, but also, *in fine*, to have a system usable for describing GTs (Figure 2) (13,16). For the classical hydrolytic activities of the GHs, the reactant is simply a water molecule, while lyases have no reactant. Hydrogen peroxide (H_2_O_2_) is the reactant for LPMOs (Table 1) (17). For phosphorylases, the reactant is inorganic phosphate, which is transferred or released, noted as ePi or aPi to indicate its equatorial or axial orientation with respect to the sugar ring (Table 1) (18). For transglycosylases, the reactant column contains the leaving part of the donor molecule, but also the anomer of the cleaved bond. For example, amylosucrase (EC 2.4.1.4), which transfers α-d-glucose onto amylose (α-(1,4)-glucan) from sucrose as a donor substrate (Figure 2). The same system will be used in the future to describe the mechanism of GTs. Finally, the three mutase functions described in family GH13 (EC 5.4.99.11, 5.4.99.15 and 5.4.99.16) are just particular transglycosylation reactions that can be described like the other transglycosylases (Figure 2).

**Table 1.** Correspondence between family class, EC numbers, activity Id and reactant

Class	EC	Activity Id	Reactant
GH			
Hydrolases	3.2.1.*x*	**H** *x*	H_2_O
Phosphorylases	2.4.1.*x* 2.4.99.*x*	**T** *x*	a/ePi
Transglycosylases	2.4.1.*x* 2.4.2.*x*	**T** *x*	a/e-leaving group
Lyases	4.2.2.*x*	**L** *x*	none
Mutases	5.4.99.*x*	**M** *x*	a/e-leaving group
PL	4.2.2.*x*	**L** *x*	none
AA-LPMO	1.14.99.*x*	**O** *x*	H_2_O_2_
GT	2.4.1.*x* 2.4.2.*x*	**T** *x*	a/e-leaving group

#### Monosaccharide description

**Figure 3.**
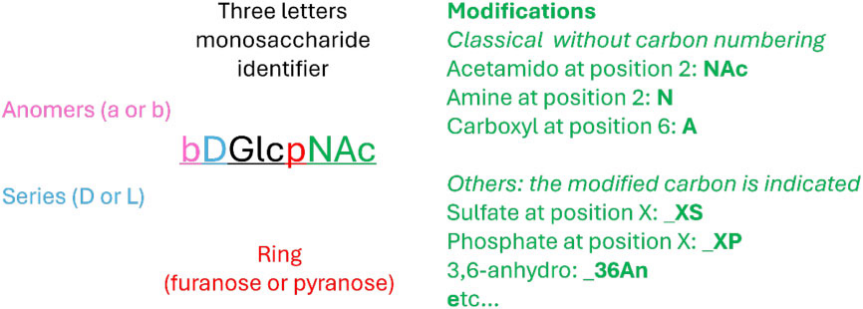
Monosaccharide nomenclature inspired by the IUPAC nomenclature of carbohydrate.

In order to implement the subsite description, a standardization and abbreviation of monosaccharides was necessary. A standardized nomenclature inspired by the IUPAC convention was devised to describe monosaccharides (Figure 3). For the simplest monosaccharides, the notation includes the anomer (α or β), stereochemistry (d or l), three letters sugar nomenclature and ring type (pyranose or furanose), e.g. β-d-glucopyranose is bDGlcp. Common modifications, like N-acetylation, are appended. For example, β-d-N-acetyl glucosamine is written bDGlcpNAc. The uncommon modifications are separated by underscore character ‘_’ and the number of the carbon modified is specified, e.g. N-acetyl galactosamine 4-sulfate found in dermatan sulfate writes bDGalpNAc_4S (Figure 3 and Table 2).

**Table 2.** Examples of residue diversity

Residue
bDGalpNAc_4S
[bDGlcpNAc
bDFruf_6P]
(aDXylp-1,6)bDGlcp
(aDGlcp)_3_
aDGlcp(cyclic)
Mandelonitrile
Alcohol

**Figure 4.**
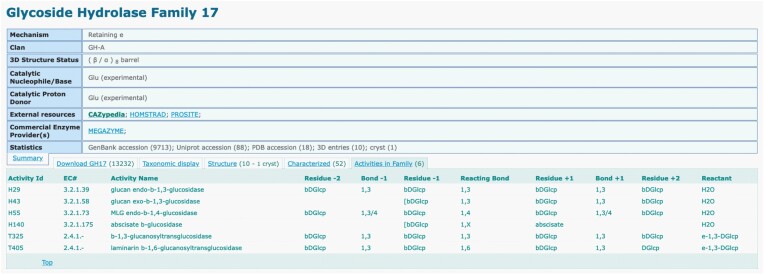
New header display in the CAZy database.

For some specific polysaccharides, a subsite may contain a monosaccharide carrying a carbohydrate side chain such as in the case of xyloglucan. The side chain and the link are indicated parenthetically before the monosaccharide, e.g. a subsite of xyloglucan is described (aDXylp-1,6)bDGlcp (Table 2). The parentheses can also be used to indicate multiple copies of the same sugar extending beyond subsite −2 or +2 (Table 2). For example, glucan 1,4-α-maltotetraohydrolase (EC 3.2.1.60), the additional monosaccharides can be added on the −2 subsite by writing (aDGlcp)_3_. Finally, a square bracket can be added to indicate the last subsite that can be occupied. This typography is particularly useful to describe NRE or RE exolytic activities (Table 2 and Figure 4). Four fields, −1 and +1 subsites, the reacting bond and the reactant are the strict minimum information to describe the simplest function of CAZymes. Regardless of the CAZyme class, the −1 subsite is the most important as it describes the basic enzymatic activity and gives GHs and GTs their names. The −1 subsite contains strictly carbohydrate information. The +1 subsite can contain a carbohydrate or a noncarbohydrate aglycon. The non-natural derivatives frequently used for enzymatic assays, such as para-nitrophenyl (pNP) groups, are described by the generic term ‘alcohol’ (Table 2). This generic term can also be used to leave the field open when the precise composition of the positives subsites is not yet identified. All physiological aglycons can be added freely, e.g. mandelonitrile to describe prunasin β-glucosidase (EC 3.2.1.118) (Table 2). The −2, and +2 subsites can be left empty, filled with monosaccharides or with any aglycon. The glycosidic bonds are simply described by the numbers of the carbons involved in the glycosidic bond, separated by a comma, e.g. ‘1,4’ (Table 3). In the case of a noncarbohydrate aglycon, or if uncertain, ‘*X*’ can be used to complete the bond, ‘1,*X*’. The specificity of LPMOs implies that the carbon of the glycosidic bond that is oxidized during the cleavage must be indicated. This information is given on the bond by the addition of ‘o’. When the C1 is oxidized, the bond is ‘1o,4’ while it is ‘1,4o’ for C4 oxidation (Table 3).

**Table 3.** Examples of bond description

Bond	Description
1,4	Glycosidic bond between C1 and C4
1,3/4	Glycosidic bond between C1 and C3 or C4
1,*X*	Glycosidic bond between C1 and an unspecified aglycon
1o,4	C1 oxidation during LPMO cleavage

All EC numbers of GHs, PLs and LPMOs, including incomplete ones lacking four-digit specificity, were translated into the CAZac system, associated to an activity ID composed of a letter and a number. The letter informs about the class of enzymatic activity, H for hydrolysis, L for lyase, T for transferase, M for mutase and O for oxidase (Table 1). The transferase class currently encompasses only phosphorylases and transglycosylases, but will be extended to GTs in the future.

#### Exceptions not covered by CAZac

Currently, 407 activities were created for the glycan-cleaving enzymes by CAZy. Only four EC were not translated to activity. The four missing activities are particular lyase activities involved in the metabolism of the difructose dianhydrides (4.2.1.179 and 4.2.2.16, 17 and 18). These reactions do not follow the classical β-elimination of the PLs, and cyclic disaccharides are formed by condensation, usually after the cleavage of the polysaccharides (19–22). The exact description of two sequential reactions could not be easily imported into the CAZac system. Although these four EC numbers are not included in CAZac, they will continue to be listed in CAZy.

## Implementation of CAZac in the CAZy website

### Implementation of the family headers

In the CAZy database, activities were previously simply listed in the header of each GH family, by their function names and the corresponding EC number, making analysis difficult and detailed comparisons impossible. Now, the listing of activities appears just below the header. The full detailed list of activities in the family is displayed with the eight fields described above, along with the EC number and the activity ID (Figure 4). For the families divided in subfamilies, the selection of a subfamily of interest limits the display to activities found in that subfamily. This new representation provides a direct access to useful information for assessing the functional diversity within a family, or subfamily.

### Search tool

**Figure 5.**
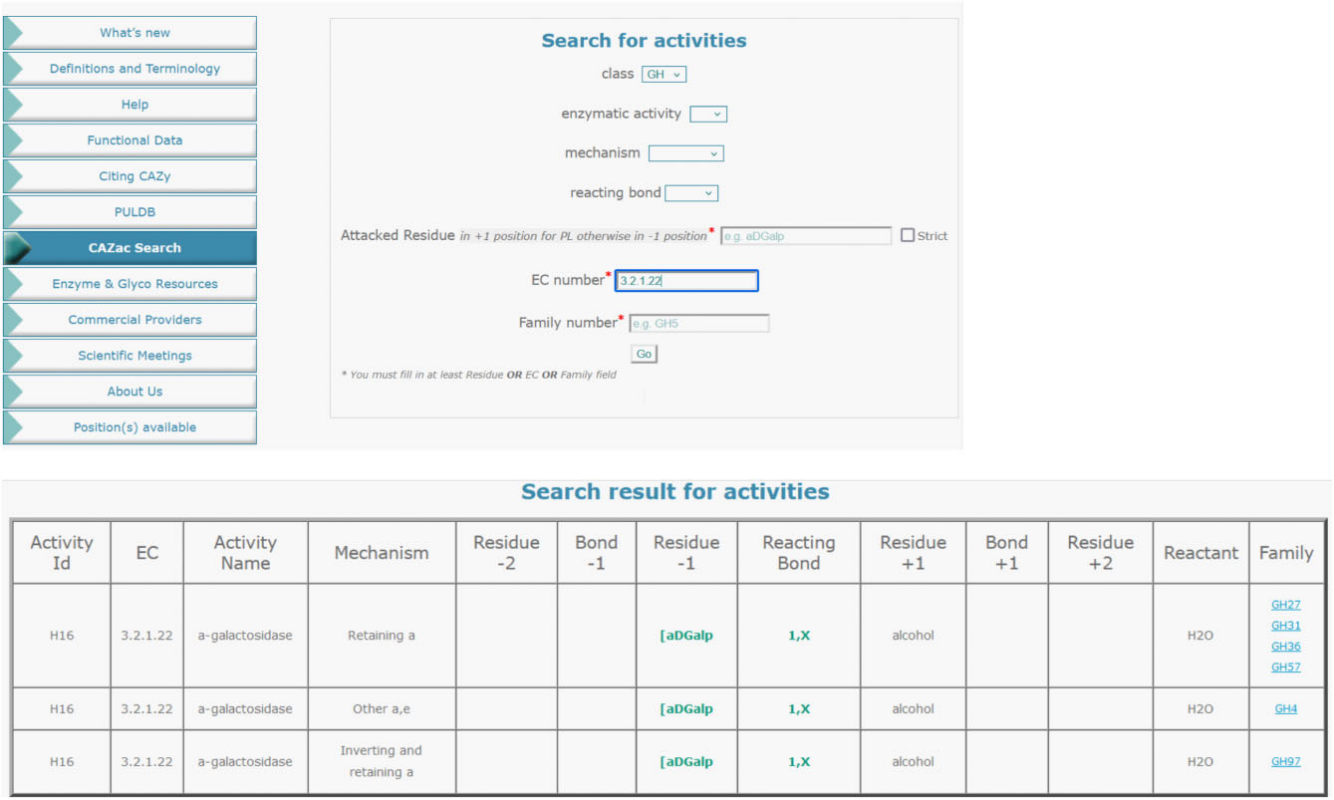
Display of the CAZac search tool, as input the EC 3.21.22 and the result table.

To enable complex requests, a search tool is now accessible via the ‘CAZac search’ tab on the CAZy main page. Seven search fields enable different combinations of searches (Figure 5). In addition to specifying the CAZyme class (H, L, T, M or O), the minimum requirement for a search is to specify the residue in the −1 subsite for GHs and LPMOs, or +1 for PLs, or an EC number or a CAZy family number. These fields are indicated by an asterisk (Figure 5). All other fields are optional and additive, i.e. the results are more restricted when more fields are defined, allowing a broad or a very specific search. The description of the monosaccharides must follow the nomenclature explained above. However, a general input such as ‘bDGalp’, retrieves activities on β-d-galactopyranose and all its variants, such as NAc, sulfation, etc. A ‘strict’ option can be added to limit the search to one specific monosaccharide, e.g. ‘strict’ bDGalp does not give activities on bDGalpNAc. In addition, the strict search on ‘[bDGalpNAc’ allows direct access to exolytic families. The field ‘enzymatic activity’ restricts the search to specific types of catalysis, such as hydrolysis (H), transferase (T) or lyase (L) within GHs families, for example. The ‘mechanism’ field finds all families that share the same mechanism for a particular function, such as retaining β-glucosidase. The reacting bond indicates the cleaved or the created linkage based on catalysis. The result of the search is displayed as a detailed table with all fields (Figure 5). Each line corresponds to an activity ID for a specific mechanism that allows discriminating between retaining and inverting families, for example. Therefore, the search EC 3.2.1.22, for the α-galactosidase function, gives three results, including ‘retaining *a*’ families (GH27, GH31, GH36 and GH57), the ‘other *a*,*e*’ (GH4) and ‘inverting and retaining *a*’ (GH97) (Figure 5).

### CAZac application example

The digestion of starch is probably one of the first functions of GHs to be studied (23). In fact, the three first EC numbers of the glycosylases concern amylose degradation: α-amylase 3.2.1.1, β-amylase 3.2.1.2 and glucan 1,4-α-glucosidase 3.2.1.3. But now, with >200 official EC numbers, the repertory of all activities against amylose or closely related substrates is complex. By searching GH in class, the reacting bond ‘1,4’ and ‘aDGlcp’ as −1 residue ‘strict’, all endo acting activities and families were found. Seventeen activities IDs divided among eight GH families act on aDGlcp-1,4 bond (Table 4). The composition of subsites confirms that ‘aDGlcp-1,4’ is mainly found in starch, only four activities are on close substrates: pullulan (H42 and H108), maltotrehalose (H112) and mycodextran (H46). In addition to degrading activities, GH families involved in starch synthesis are also found: one phosphorylase (T292) and three transglycosylation activities (T309, T311 and T338). The family diversity shows that GH13 is the most dominant family, with 13 of 17 activities. This search can be completed by searching the exo active enzymes. The same request is done by adding a square bracket before the −1 residue description ‘[aDGlcp’. Seven additional activities are found, namely two hydrolases, one phosphorylase, three transglycosylases and one lyase (Table 5). As for endo activities, exo activities are mainly against starch or malto-oligosaccharides.

**Table 4.** Results for the endo acting enzymes on ‘aDGlcp-1,4’

Activity Id	EC	Activity name	Mechanism	Res −2	Bond −1	**Res −1**	**Reac bond**	Res +1	Bond +1	Res +2	Reactant	Family
Hydrolases												
H1	3.2.1.1	amylose endo-a-1,4-glucosidase	Retaining a	aDGlcp	1,4	**aDGlcp**	**1,4**	aDGlcp	1,4	aDGlcp	H2O	GH119
												GH13
												GH57
			Inverting (inferred) a	aDGlcp	1,4	**aDGlcp**	**1,4**	aDGlcp	1,4	aDGlcp	H2O	GH126
H2	3.2.1.2	amylose exo-a-1,4-glucobiosidase	Inverting a	[aDGlcp	1,4	**aDGlcp**	**1,4**	aDGlcp	1,4	aDGlcp	H2O	GH14
H40	3.2.1.54	cyclomaltodextrin endo-a-1,4-glucosidase	Retaining a	aDGlcp	1,4	**aDGlcp**	**1,4**	aDGlcp	1,4	aDGlcp(cyclic)	H2O	GH13
												GH57
H42	3.2.1.57	pullulan exo-a-1,4-isopanosidase	Inverting a	[(aDGlcp-1,4)aDGlcp	1,6	**aDGlcp**	**1,4**	aDGlcp	1,4	aDGlcp	H2O	GH49
H45	3.2.1.60	amylose exo-a-1,4-glucotetraosidase	Retaining a	[(aDGlcp)3	1,4	**aDGlcp**	**1,4**	aDGlcp	1,4	aDGlcp	H2O	GH13
H46	3.2.1.61	mycodextran endo-a-1,4-glucosidase	Unknown a	aDGlcp	1,4	**aDGlcp**	**1,4**	aDGlcp	1,3	aDGlcp	H2O	GH87
H75	3.2.1.98	amylose exo-a-1,4-glucohexaosidase	Retaining a	[(aDGlcp)5	1,4	**aDGlcp**	**1,4**	aDGlcp	1,4	aDGlcp	H2O	GH13
H91	3.2.1.116	amylose exo-a-1,4-glucotriosidase	Retaining a	[(aDGlcp)2	1,4	**aDGlcp**	**1,4**	aDGlcp	1,4	aDGlcp	H2O	GH13
H107	3.2.1.133	amylose exo-a-1,4-glucobiosidase	Retaining a	[aDGlcp	1,4	**aDGlcp**	**1,4**	aDGlcp	1,4	aDGlcp	H2O	GH13
												GH57
H108	3.2.1.135	pullulan exo-a-1,4-panosidase	Retaining a	[(aDGlcp-1,6)aDGlcp	1,4	**aDGlcp**	**1,4**	aDGlcp	1,4	aDGlcp	H2O	GH13
H112	3.2.1.141	maltotrehalose a-1,4-glucosidase	Retaining a	aDGlcp	1,4	**aDGlcp**	**1,4**	aDGlcp	1,1	aDGlcp]	H2O	GH13
H182	3.2.1.-	amylose exo-1,4-glucopentaosidase	Retaining a	[(aDGlcp)4	1,4	**aDGlcp**	**1,4**	aDGlcp	1,4	aDGlcp	H2O	GH13
H249	3.2.1.-	panose a-1,4-glucosidase	Inverting a	aDGlcp	1,6	**aDGlcp**	**1,4**	aDGlcp	1,4	aDGlcp	H2O	GH49
Phosphorylases												
T292	2.4.99.16	starch phosphorylase	Retaining a	[aDGlcp	1,4	**aDGlcp**	**1,4**	aDGlcp	1,4	aDGlcp	aPi	GH13
Transglycosylases												
T309	2.4.1.19	cyclomaltodextrin glucanotransferase	Retaining a	aDGlcp	1,4	**aDGlcp**	**1,4**	aDGlcp	1,4	aDGlcp(cyclic)	a-1,4-aDGlcp	GH13
T311	2.4.1.25	4-a-glucanotransferase	Retaining a	aDGlcp	1,4	**aDGlcp**	**1,4**	aDGlcp	1,4	aDGlcp	a-1,4-aDGlcp	GH13
												GH57
												GH77
T338	2.4.1.-	a-maltosyltransferase	Retaining a	[aDGlcp	1,4	**aDGlcp**	**1,4**	aDGlcp	1,4	aDGlcp	a-1,4-aDGlcp	GH13

The residue important for the reaction and the reacting bond are indicated in bold.

**Table 5.** Results for the exo acting enzymes on ‘aDGlcp-1,4’

Activity Id	EC	Activity name	Mechanism	Res −2	Bond −1	Res −1	Reac bond	Res +1	Bond +1	Res +2	Reactant	Family
Hydrolases												
H3	3.2.1.3	amylose exo-a-1,4-glucosidase	Inverting a			**[aDGlcp**	**1,4**	aDGlcp	1,4	aDGlcp	H2O	GH15
			Inverting and retaining a			**[aDGlcp**	**1,4**	aDGlcp	1,4	aDGlcp	H2O	GH97
H407	3.2.1.-	maltose a-1,4-glucosidase	Retaining a			**[aDGlcp**	**1,4**	aDGlcp]			H2O	GH13
Phosphorylases												
T265	2.4.1.8	maltose phosphorylase	Inverting a			**[aDGlcp**	**1,4**	DGlcp]			ePi	GH65
Tranglycosylases												
T304	2.4.1.4	amylosucrase	Retaining a			**[aDGlcp**	**1,4**	aDGlcp	1,4	aDGlcp	a-1,2-bDFruf]	GH13
T318	2.4.1.161	oligosaccharide 4-a-D-glucosyltransferase	Retaining a			**[aDGlcp**	**1,4**	aDGlcp	1,4	aDGlcp	a-1,4-aDGlcp	GH13
												GH31
T330	2.4.1.-	reuteransucrase a-1,4-glucosyltransferase	Retaining a			**[aDGlcp**	**1,4**	aDGlcp	1,2	bDFruf]	a-1,2-bDFruf]	GH70
Lyases												
L345	4.2.2.13	Exo-1,4-a-D-glucan lyase	Retaining a			**[aDGlcp**	**1,4**	**aDGlcp**	1,4	aDGlcp		GH31

The residue important for the reaction and the reacting bond are indicated in bold.

## Discussion

The huge natural glycan diversity is paralleled by a similar diversity of the enzyme activities involved in their synthesis and their breakdown. Since 1998, the CAZy database presents a continuously updated sequence-based classification of CAZymes. A major on-going effort is for collecting functional information from the literature to enrich the database (2). However, <1% of sequences in CAZy (all enzyme classes combined) have been functionally characterized. This huge and fast-growing gap results from the technical difference between automated large-scale DNA sequencing and functional characterization, which is still limited by experimentation, such as protein expression, solubility or stability. For CAZymes, substrate availability for assays is an additional issue. Unfortunately, many if not most CAZyme substrates are not yet known or not commercially available, and only a small number can be sourced. Consequently, CAZymes assays are often limited to nonphysiological substrates such as pNP-sugars or synthetic cellulose derivatives, e.g. carboxymethylcellulose, hydroxyethylcellulose, etc. This substrate limitation restricts our ability to explore and harness the diversity of CAZymes. Efforts to circumvent these limitations will be long and the development of functional predictions could be a good alternative. However, predictive approaches require a system that allows functional data to be accurately described and easily searched, exploited and compared, which is difficult with the EC classification. Because it combines accurate function description and reaction mechanisms, our activity descriptor, CAZac, allows searches within a family as well as across all CAZyme families. By making it possible to inspect closely related families, the CAZac system will also help guide the search for new CAZyme activities.
